# Supplementary material for: Are carbon emissions trading and green financial instruments synergistic? -Comprehensive quantitative research based on content analysis
Source: PLoS One. 2024 Mar 7;19(3):e0298601. doi: 10.1371/journal.pone.0298601 (PMC10919601; doi:10.1371/journal.pone.0298601)
Supplement: S1 Data — (ZIP) [file pone.0298601.s001.zip › Data/地级市Z_F工作报告.zip!/╝¬┴╓/╦─╞╜╩╨/└┤╫╘╣1⁄2╓┌║┼í╛┬φ┐╦╩2╛▌═°í┐_2009.docx]

各位代表：

　　现在，我代表市人民政府，向大会报告工作，请予审议。

　　一、2008年工作回顾

　　2008年，我们在省委、省政府和市委正确领导下，着力推进四平大发展、快发展，在上半年国家宏观调控趋紧，下半年受到金融危机冲击的形势下，积极采取措施，克服各种困难，继续保持了全市经济社会发展的良好势头。

　　——国民经济快速健康发展。预计全年GDP实现585亿元，同比增长21%，其中一、二、三产业增加值实现195亿元、201亿元、189亿元，同比分别增长7.5%、25.6%和17.5%，三次产业比重调整到33.3：34.4：32.3。预计全年规模以上工业企业总产值实现520亿元，同比增长40%，固定资产投资完成339.5亿元，同比增长53.8%，增幅在全省领先。全市一般预算全口径财政收入预计实现37.6亿元，同比增长30.4%，地方级财政收入实现16.9亿元，同比增长36.5%，其中，市本级一般预算全口径财政收入实现19.9亿元，同比增长30%，地方财政收入实现7.1亿元，同比增长40.2%；全口径财政支出59.2亿元，同比增长15.3%，其中市本级财政支出19.8亿元，同比增长18%。全市进出口总额预计实现1.45亿美元，同比增长40.2%。社会消费品零售总额预计实现198亿元，同比增长25%。居民消费价格指数控制在105.9%。顺利实现市委提出的“一个巩固、两个领先”目标，圆满完成市六届人大一次会议确定的各项任务。

　　——工业经济提速增效明显。工业主要指标继续保持快速增长势头，规模以上工业企业达到372户，预计实现增加值175亿元，同比增长27%；实现利润15亿元，同比增长47%。红嘴集团、四平烟厂、双辽电厂等重点企业对全市工业经济的支撑和拉动作用不断加大。民营经济不断发展，主营业务收入预计实现536亿元，同比增长35.1%。节能降耗取得新成效，全市单位GDP能耗预计下降4.7 %，单位工业增加值能耗下降8%。

　　——重点项目扎实推进。始终把项目建设作为经济工作的重中之重，集中力量做项目、跑项目、争项目。全市3000万元以上在建项目达到330个，其中亿元以上项目130个。全年共争取到国家预算内投资项目89项，争取到国家投资1.33亿元。在国家新增1000亿元扩大内需投资中，我市共争取9大类59项，资金3.74亿元，排在全省前列。四平烟厂新型卷烟、金士百80万吨啤酒、昊华“双20万吨”离子膜烧碱和聚氯乙烯、中粮黄龙65万吨玉米深加工、博来德30万吨大豆加工项目按期竣工投产，莫里青油田开发项目油井总数达到140口，产能达到21.5万吨。庆达30MW非晶硅、吉林世宝精密铸造机加项目、巨元比克换热设备园区、吉云鹿业梅花鹿精深加工、玛钢年产100万台（套）轿车发动机飞轮、华翔工业园、高金食品年产5万吨猪制品、天丰科技50万吨马铃薯资源循环及综合利用及30万吨复合肥、红嘴曙光牧业2000万只肉鸡屠宰等超亿元大项目正式开工建设。天成60万吨玉米深加工及100万吨粮食物流、梨树新天龙8万吨醋酸、公主岭2万吨木糖醇、华翔汽车内饰件等一批续建项目进展顺利。四平热电厂二期和双辽电厂二期扩建项目前期工作全部完成。存量资产进一步盘活。原东风四平客车公司被红嘴集团收购改造，年产300辆大客车部分整车下线投放市场；东风收获机械有限公司与中国一拖集团正式签订合作协议；纳爱斯四平公司推出新产品，扩大了产能。

　　——招商引资成效显著。坚持高位运作，不断创新招商引资方式方法，实施重点领域招商，提高针对性和实效性。积极赴日本、俄罗斯、深圳、南京、江西等国家和省（市）进行经贸交流活动，组织参加东博会、农博会等各种展会和洽谈会，取得了良好效果。全年引进项目729个，续建项目237个，实际到位资金234.6亿元，同比增长50.1%。开发区、工业集中区对外开放和吸引项目能力进一步增强。我市被评为“中国最具投资潜力中小城市二十强”。

　　——农村经济全面发展。粮食产量达到135亿斤，获得空前大丰收。畜牧业继续扩张，全市猪、牛、羊、禽饲养量预计分别达到1100万头、270万头、190万只和1.7亿只。农业产业化经营取得新进展，规模以上农产品加工企业发展到169户，中粮黄龙、天成玉米、红嘴农高等一批重点龙头企业带动和辐射能力不断增强，全市农产品加工企业实现产值160亿元、利润6.6亿元，同比分别增长18.5%、15%。园艺特产业快速发展，形成了梨树棚膜蔬菜、双辽花生、伊通鹿业等特色产业基地。以红嘴牧业合作社为代表的农村各类合作经济组织发展到683个，经纪人队伍达到9000多人。新农村建设稳步推进，全市四级试点村镇达到289个。县域基础设施建设取得新成效，公路建设竣工里程917公里，伊通至辽源高速公路四平段完成年度计划，国道303线双辽段建成通车，农村公路竣工880公里，行政村通油路（水泥路）率达到81.3％。农村泥草房改造完成20274户，超额完成省下达任务。劳务经济健康发展，转移农村劳动力51.4万人（次）。农村体制改革不断深化，集体林权制度改革试点进展顺利，国有粮食购销企业改革扎实推进，农行面向“三农”金融服务试点工作深入开展，发放“惠农卡”14万张，放款5亿元。

　　——城市建设管理取得新成绩。全市棚户区改造预计完成拆迁面积99万平方米，完成施工面积126万平方米。城镇低收入住房困难家庭住房保障制度全面建立，租赁住房补贴和实物配租工作已经展开。市区新增集中供热能力110万平方米。新建、翻建和维修市区道路21条，东山大街、平东公铁立交桥建成通车。城区卫生环境综合整治会战取得实效，保持了国家卫生城荣誉，城市园林绿化取得新成果，成功创建国家园林城市。加强住宅小区及棚改小区管理，市区1413栋楼房的黑楼道全部亮化。实施重点减排工程，城市垃圾焚烧发电项目、伊通污水处理厂开工建设，市区、公主岭、梨树、双辽等4个污水处理厂竣工投产。坚持污染防治与生态保护并重，强化环境监督管理，全市化学需氧量和二氧化硫排放量分别削减2770吨和1084吨。城市适宜居住和创业的功能不断增强。

　　——民生问题得到较好解决。认真抓好就业再就业，全市预计新增就业人员5.4万人，下岗失业人员再就业4.4万人，城镇登记失业率控制在4%以内，城镇劳务输出预计达到58.2万人（次）。城镇居民年人均可支配收入实现13600元，同比增长9.7%；农民年人均纯收入实现4600元，同比增长7.5%。进一步扩大社会保险覆盖面，城镇基本医疗、养老、失业保险参保人数分别达到74.4万人、34万人和20.7万人，养老金社会化发放率、及时足额发放率达到100%，新征地农民养老保险政策开始实施。城乡有12.4万和3.3万低保对象得到保障，年发放低保金分别达到1.7亿元和4612万元。为245户符合条件的国有改制破产企业发放了退休职工取暖费补贴。积极促进教育公平，义务教育阶段学杂费全部免除。加强食品、药品市场监管，保障群众饮食和用药安全。建设农村饮水安全工程85处，解决了4.62万人饮水困难问题。全面规范企业用工行为，维护劳动者合法权益。积极开展扶贫帮困和灾民救济，困难群众得到及时救助。

　　——社会事业协调发展。走科技兴市之路，全市有25个项目列入国家、省科技发展计划，省级以上高新技术企业达到61户，年产值增长率达到35%。促进教育均衡协调发展，全年投入义务教育保障经费1.33亿元，完成农村初中校舍标准化改造9561平方米，大力发展职业教育，建成国家级重点职业学校3所。着力推进农村乡镇卫生院改造和城市社区卫生服务体系建设，改造乡镇卫生院65个，新建社区卫生服务中心13个。全市参加新型农村合作医疗的农民达到169.3万人，参合率达到82.2%。切实加强公共卫生、医疗服务管理和中医药工作，医疗卫生服务条件不断改善，中心医院新门诊大楼、第一医院综合大楼投入使用。积极探索开展生育关怀的有效形式，各项生育关怀政策得到落实，全国、全省生育关怀现场会推广了四平经验。群众性文化活动丰富多彩，成功举办了纪念四平解放60周年系列文艺演出活动。积极推进广播电视“村村通”工程，广播电视综合覆盖质量不断提高。群众体育蓬勃开展，竞技体育再创佳绩，四平籍运动员在29届北京奥运会上夺得1枚金牌、2枚银牌。旅游产业发展迈上新台阶，牧情谷景区和伊通满族博物馆被国家命名为4A级景区景点。加大安全生产监管力度，年初以来未发生重特大安全事故。深入开展平安四平创建工作，保持了社会治安形势稳定。双拥活动深入开展，军政军民关系进一步密切。金融、知识产权、民族宗教、外事、侨务、人防、地震、气象、老龄、地方志和残疾人等工作取得新的成绩。

　　——精神文明和民主法制建设进一步加强。文明城市创建活动扎实推进，思想道德建设得到加强。深入开展“‘树新风正气，促和谐发展’，从我做起”主题教育活动，机关作风有了新的转变。大力开展为汶川地震灾区募捐活动，共募集款物价值4090.2万元。自觉接受人大法律监督、工作监督和政协民主监督。全年办理人大代表建议124件，政协委员提案229件，办复率达100%。加强政府法制工作，坚持依法行政。支持审计部门开展审计监督。落实信访责任制和领导信访接待制度，认真解决群众反映的突出问题，实现了信访总量下降和奥运期间非正常进京访零登记。

　　各位代表，这些成绩的取得，是市委正确领导的结果，是全市各族人民共同努力的结果，是社会各界大力支持的结果。在此，我代表市人民政府，向奋战在全市各条战线的广大劳动者，向给予政府工作热情支持的人大代表、政协委员、各民主党派、工商联、各人民团体和社会各界人士，向关心帮助四平建设的驻平部队、武警官兵、中省直单位及海内外朋友表示崇高的敬意和衷心的感谢！

　　在看到成绩的同时，我们也清醒看到，我市经济和社会事业发展中还存在一些矛盾和问题，主要是发展基础还不牢固，经济总量不大、结构不优、效益不高，自主增长、良性循环机制尚未完全形成；财政支撑保障能力不强，财力增长远远低于刚性支出的增长，收支矛盾十分突出，尽管下力量解决了一些问题，但和人民群众要求还有差距；现代服务业发展滞后，与一、二产业衔接不够紧密，相互促进作用没有发挥出来；农业基础设施薄弱，发展现代农业、推进新农村建设需付出艰苦努力；改善民生特别是增加城乡群众收入、就业再就业、社会保障压力较大，维护社会稳定和化解矛盾的任务繁重；部分干部思想观念、工作作风与新形势、新任务的要求还不适应。这些问题需要在今后的工作中认真解决。

二、2009年工作安排

　　2009年是充满挑战的一年。国际金融危机对实体经济造成的冲击逐步显现，危机何时见底，对我们影响会有多大，一时还难以预料，形势严峻。同时也要看到，中央实施积极的财政政策和适度宽松的货币政策，扩大内需，加大投资力度，我市作为粮食主产区和投资拉动型经济，将受益更多；我市正处在工业化、城镇化推进阶段，发展空间大，特别是基础设施建设潜力大；沿海地区新一轮经济结构调整和产业转型，给我们承接产业转移和招商引资带来新的契机；四平近几年经济社会快速发展，为今后加快发展奠定了良好基础。我们要准确把握形势，坚定必胜信心，变压力为动力，化挑战为机遇，迎难而上，抢占先机，继续保持四平发展的好势头，开创全市经济社会发展新局面。明年工作总体思路是：以党的十七大和十七届三中全会精神为指导，认真贯彻中央经济工作会议精神，深入落实科学发展观，立足保持经济平稳较快增长，继续推动大上项目、全民招商、全民创业，深入实施工业立市、科教兴市和县域突破战略，加大投资拉动力度，保增长、扩内需、调结构，引领经济社会大发展、快发展，着力解决民生问题，为完成“一个巩固、两个领先”任务，早日实现四平振兴、人民富裕而努力奋斗。

　　主要预期目标是：全市完成生产总值681亿元，增长16.4%；一般预算全口径财政收入完成43.2亿元，增长15%；全社会固定资产投资完成465亿元，增长37.1%；规模工业完成总产值618亿元，增长20％，增加值200亿元，增长12.3％；社会消费品零售总额实现238亿元，增长20%；外贸进出口总额完成1亿美元；城镇居民人均可支配收入实现14960元，农民人均纯收入实现4960元，分别增长10%和8%；居民消费价格指数为104%；新增城镇就业5.3万人，城镇登记失业率控制在4.5%以内；人口自然增长率控制在5‰以内；万元GDP能耗下降4.5%。

　　为实现上述目标，重点做好以下工作，力争超额完成任务：

　　（一）突出项目带动，全力培育新的经济增长点

　　紧紧抓住国家实施积极的财政政策和适度宽松的货币政策的新机遇，超前准备，加大力度，集中力量抓好一批关系全局的重大建设项目。预备项目抓前期。认真做好四平烟厂30万大箱卷烟异地改造、四平至长春高速公路双向8车道拓宽改造、集双高速公路四平段、红嘴200万吨钢扩建、公主岭60万千瓦热电厂、煌上煌1000万只家禽屠宰等项目的前期工作，尽快报批。新上项目抓开工。加速推进天成15万吨L?乳酸和5万吨聚乳酸及12万吨生物丁醇、精细化工100吨头孢西丁酸、长江客车1万台高档环保旅游客车、梨树生物质热电厂、和颐工业园、伊通金圆水泥二期等新项目，促其尽快开工建设。在建项目抓进度。全力推进哈大客运专线、庆达30MW非晶硅薄膜太阳能电池、吉林世宝高强度球铁毛坯件制造、东风机械5000台纵轴系列收割机等在建项目的建设进度。计划投产项目抓达效。继续抓好吉春制药“十味降糖颗粒”及“妇炎康复胶囊”、天丰科技50万吨马铃薯循环利用及30万吨有机、无机复合肥、公主岭2万吨木糖醇、华翔工业园等计划投产项目的建设，确保年内投产达效。全面包装争取项目，根据国家政策方向和资金投向，围绕我市主导、优势产业，以做大做强重点企业、培育新的经济增长点、扩大就业容量为目标，精心谋划、论证、上报一批带动能力强、成长性好、附加值高的大项目、好项目，把项目前期工作做足做透，加强储备，保证项目接续。保障项目用地，节约、集约、规范使用土地，提高投资强度。

　　着力提高招商实效性和成功率。利用四平区位、企业、资源、市场等优势，主动与大企业、大集团寻求合作，引进资金、技术和先进的管理理念，帮助本地企业提高产品质量，扩大生产规模，延伸产业链条。搞好石油、天然气、膨润土、油砂、褐煤等资源类项目的研制、包装和开发，打造资源新优势。紧盯长春、沈阳和沿海发达地区，主动承接产业转移和辐射带动。制定优惠政策，建立激励机制，增强全民招商意识，形成全民招商热潮。全年完成招商引资任务300亿元，同比增长30%。

　　发挥开发区、工业集中区项目建设先导作用。做好科学定位，进一步完善开发区、工业集中区发展规划。在特色园区建设上实现突破，努力打造汽车零部件、光伏产业、绿色食品、换热器等特色鲜明的产业园区，尽快形成产业集群。在大项目开发上实现突破，每年都要有一批超亿元项目进区。在基础设施建设上实现突破，通过向上争取和市场化运作筹集建设资金，尽快实现“七通一平”。搞好红嘴大学科技园、换热器国检中心、四平经开区中小企业科技园建设，整合资源，培育孵化项目。推进开发区、工业集中区体制机制创新，在运行机制、经营模式、干部管理、奖惩措施等方面拿出办法，打造发展新优势。

　　（二）调整优化结构，进一步发挥工业主导作用

　　坚持工业立市不动摇，采取措施积极应对国际国内各种不利因素挑战，促进产业优化升级，推进工业提速增效。加快发展主导产业，提升机械加工制造业的规模和实力，延伸农副产品加工业产业链条，开发高附加值终端产品。全力做大优势产业，积极推动能源、冶金、化工、建材产业优化升级。扶优扶强。大力支持红嘴集团、四平烟厂、双辽电厂、四平电厂、黄龙、昊华、天成、收割机、巨元比克、庆达光盘、精细化工等重点企业发展壮大。大力推进企业自主创新。实施技改“双百”工程，技术改造项目超百项、技改投资达到百亿元，提高企业装备水平和产品市场竞争力。加快推进企业上市工作。选择资产优良、成长性好的企业，抓紧进入辅导期，择机上市融资，实现上市公司零的突破。改善金融生态环境。促进企业、银行、担保公司对接制度化、常态化，拓宽融资渠道，用好开行贷款和其它商业银行贷款，吸纳域外资金和民间投资，实现项目投资主体多元化。大力实施品牌战略。培育和创造具有四平地域特色的名牌产品和驰名商标，明年创中国名牌2个，省名牌12个。认真落实节能减排责任制，提高能源利用效率。加强工业经济运行的综合协调，搞好生产要素供需衔接。

　　（三）统筹城乡发展，加快农村改革发展步伐

　　发挥农业比较优势，强化农业基础地位，推进农村改革创新，加快发展县域经济，形成城乡经济社会发展一体化新格局。大力发展现代农业，着力提高粮食综合生产能力，加快推进增产30亿斤粮食工程。加快实施大中型灌区改造、中小河流治理、病险水库除险加固等水利项目，做好中部城市引松供水四平段工程前期工作。积极争取农机具购置补贴，鼓励农户购买大型农机具，新增农机动力10万千瓦。加快农业科技创新，推广应用8项先进适用技术。加快发展园艺特产业，抓好10万亩棚膜经济园区，扩大和提升鹿产品加工规模和档次。大力发展畜牧业，新建牧业小区100个，打造公主岭和梨树双1000万头生猪饲养繁育基地，形成饲养、加工、经营一体的产业集群，建设牧业强市。不断优化农业产业结构，做强农产品加工业，加快500万吨粮食加工基地和1000万头生猪加工能力建设。积极推广红嘴农村专业合作社的经验，提高农民生产经营组织化程度，增强市场应变能力。加强农村清洁能源、电网改造、广播电视、信息服务等公共设施建设，新建农村公路1000公里，改善农民生产生活条件。发挥城镇辐射带动作用，在交通便利的公路沿线按照工业强镇、商贸大镇、旅游名镇、民族集镇等不同特点，超前规划包装20个特色鲜明重点城镇，争取得到省里资金扶持。同时，在现有一些特色村基础上，打造50个重点村。稳定和完善农村基本经营制度，发展多种形式适度规模经营。加快集体林权制度改革，促进现代林业发展。抓好农村融资体制改革，搞好村镇银行、贷款担保公司和农村资金互助社试点，大力发展小额信贷，活化农村资金。全面落实各项强农惠农政策，促进农业发展、农民增收、农村繁荣。

　　（四）提升产业层次，大力发展第三产业和民营经济

　　把第三产业和民营经济作为拉动经济增长的重要力量，着力提高发展层次和水平。加快发展旅游产业。编制完成《四平市旅游产业发展总体规划》，做好创建国家优秀旅游城市基础工作，加快中心城市周边景区景点和休闲旅游度假服务区建设，深度开发叶赫满族风情旅游带，推进世界萨满生态城建设，培育精品旅游线路，着力打造特色旅游景观。大力发展现代服务业。重点围绕装备制造业搞好外包服务、围绕高速路网搞好生活服务；壮大区域内商业批发和物流龙头企业，建设具有区域特色的城乡批发市场，规划和培育装备制造业物流中心、农机物流中心和农产品物流中心。积极发展资产评估、行业协会、咨询等中介组织和金融、保险、信息、会展等新兴服务业。稳定发展房地产业。落实提高住房公积金贷款额度、税费减免等政策，刺激购房需求，加强房地产市场调控，促进房地产市场健康、有序发展。

　　继续推进民营经济腾飞。加快民营企业股份制改造，引进风险资本，加强民营企业融资平台和中小企业服务体系建设，拓宽民营企业融资渠道；引导成长型中小企业在提高经济效益的基础上，增加积累和科技投入，提升发展层次。激发全民创业热情，落实各项奖励政策和帮扶措施，调动百姓创家业的积极性，大力支持机关事业单位人员领办创办实业，鼓励在外人员返平创业。加强创业能力建设，制定培训计划，通过组织参加各种研修班以及考察、培训等活动，不断增强管理、融资、营销和研发能力，提高全民创业指数。明年全市产值超2亿元的民营企业达到45户，新增5户，规模民营工业企业达到300户，新增40户，民营经济主营业务收入、上缴税金等指标比2006年翻一番。

　　（五）坚持建管并重，努力开创城市建设和管理新局面

　　以打造最适合人居、最适合创业、最适合发展城市为目标，高标准、高质量地搞好城市总体规划的修编，完成现有城市规划区控制性详规的编制。全力巩固国家卫生城、国家园林城成果，加快城市基础设施建设步伐，加强城市管理，搞好城市净化、绿化、亮化、美化工程，提升城市品位。城市建设主要抓好六件事：一是抓好市区道路改造工程，建设东丰路、北兴平街、新植物园东路等25条主次街路。二是开工建设仁兴街、兴红街两座公铁立交桥。三是加快建设生活垃圾处理厂、新加坡不夜城，开工建设体育中心，启动中水回用工程，完成广电大厦及市区污水截流干管收尾工程。四是继续抓好棚户区改造和危旧房屋改造，完成拆迁70万平方米，建设面积100万平方米，妥善安置动迁居民。五是加大园林绿化设施建设，启动建设勤富水上公园、东山生态植物园、勤业公园。六是以一经街亮化为重点，全面提升市区的亮化水平，用两年时间，完成市区所有次干路路灯照明工程。

　　（六）发展社会事业，促进社会全面进步

　　大力发展教育、科技事业，依靠科技进步和提高劳动者素质推动经济发展。办好各级各类教育，大力发展职业教育特别是农村中等职业教育，扎实推进素质教育，促进教育均衡协调发展。发挥科技对经济发展的支撑作用，构建产学研相结合的科技创新体系。积极申办省级可持续发展实验区，争取国家和省科技创新基金；加强与北京中关村园区的对接交流，力争引进172项高新技术成果；整合市内科研力量，实现人才、设备、信息资源共享。加强人力资源开发，建立交流储备平台，重要岗位实行雇员制，使更多各类优秀人才汇集四平。加强对自主知识产权的开发、保护和利用，建立奖励基金，鼓励发明创造。完善城乡公共卫生体系，加强城市社区卫生工作，加快农村医疗卫生事业发展，建设农村三级公共卫生服务网络，切实增强农村基本公共医疗卫生服务能力。积极发展文化事业，筹办国庆60周年系列活动。继续实施“广播电视村村通”工程，完成市区有线电视数字化整体转换。认真做好人口与计划生育工作，稳定低生育水平，提高出生人口素质。认真贯彻落实《未成年人保护法》，促进青少年身心健康。广泛开展全民健身活动，提高体育工作水平，办好第五届全省少数民族运动会。做好“双拥”工作，为部队办实事，进一步密切军地军民关系。努力做好人防、统计、民族宗教、外事、侨务、气象、地震、残疾人、老龄、地方志等工作。

　　（七）维护群众利益，扎实做好保障和改善民生工作

　　民生问题关系人民群众的切身利益，是政府一切工作的出发点和落脚点。下力量抓好就业再就业工作。搭建公共服务平台，新的人力资源市场明年内建成投入使用；加强面向全体劳动者的职业培训，搞好返城返乡务工人员培训，对“零就业”家庭、“4050”人员、城镇有劳动能力的残疾人和农村剩余劳动力实行免费培训；大力开展创业促就业，落实税费减免、发放小额担保贷款等扶持政策，城镇新增就业5.3万人，保持“零就业家庭”动态为零，最大限度拓展农村劳动力就业渠道，鼓励农民走出国门创业就业；切实维护职工合法权益，全面实施劳动合同制度，企业劳动合同签订率达到95%以上，加强劳动监察和劳动保护，营造公平就业环境。进一步完善社会保障体系。完善城镇职工基本养老保险制度、城镇职工和城镇居民基本医疗保险制度，城镇居民参加基本医疗保险达到43万人，启动医疗保险进社区工程；提高农村合作医疗统筹层次和筹资水平，逐步建立农民大病保险统筹机制；加大基本养老保险费、失业保险费的扩面征缴和清欠力度，做到应收尽收，不断提高保障能力和支付水平。健全社会救助体系。提高城乡居民最低生活保障标准，城镇低保人均月标准由143元提高到180元，农村特困人口全部纳入低保范围，人均年保障金由693元提高到1000元。积极解决中低收入家庭的居住困难问题，集中建设廉租房8万平方米，对最低收入住房困难家庭发放租赁补贴。完成农村泥草房改造1.5万户。完成市区15座二次供水泵站及支线管网改造，提高供水保障能力。投资4300万元解决农村8.5万人安全饮水问题。加大对非法排污企业的监管力度，坚决完成各项环保任务指标。强化对餐饮卫生、食品、药品安全的监管，保证广大群众吃上放心食品，用上放心药品。加强价格监管，落实好各项补贴政策，确保群众基本生活水平不因经济形势变化而下降。根据财政可承受能力，最大限度解决市直机关工作人员津贴补贴问题。

　　（八）排查化解矛盾，全面做好维护社会稳定工作

　　落实稳定工作责任制，保平安、保稳定。进一步加大信访工作力度，排查化解矛盾纠纷，提高处理信访问题的超前性、及时性、实效性，努力把问题解决在萌芽之中。进一步完善各类应急预案，加强应急值守和应急演练，妥善处置突发性事件。加强安全生产工作，严格事故隐患整改和重大危险源的监控，预防和减少安全生产事故。强化社会治安综合治理各项措施，推进城市报警和监控系统建设，实现全市重点部位、易发案区域24小时监控。进一步完善社区和农村警务建设，提高社会治安防控能力。在市区和县城推行网格式巡逻，把警力摆到街面、社区和复杂场所，防范薄弱时段，提高见警率、管事率和结案率。严厉打击各种刑事犯罪，确保人民群众生命财产安全。继续清除传销、制假售假活动，进一步整顿和规范市场经济秩序。坚决查处 “黑车”非法营运，保证运输市场健康有序发展。深入开展普法教育，增强公民法制观念。

　　（九）加强自身建设，切实提高政府执行力

　　明年是建国60周年，也是四平改革发展至关重要的一年。发展任务艰巨，肩负责任重大，人民群众对我们充满期待。必须适应新形势、新任务的要求，加强政府自身建设，切实提高执行力，努力建设人民满意的服务型政府。

　　狠抓工作落实。增强事业心、责任感，对重点工作、重点项目，只争朝夕、全力以赴，说干就干，干就干好，不达目的决不罢休。做官要做事，做事要尽责，对复杂矛盾和问题，敢于碰硬，敢于负责。转变作风，加快节奏，促进政府工作全面提速，运转更加快捷高效。加强绩效考核，建立交办、督办、催办、查办制度，确保政令畅通，执行有力，落实到位。

　　优化发展环境。强化服务意识，为投资者和创业者排忧解难，营造“重商、亲商、扶商”的良好氛围。进一步简政放权，减少收费，降低项目建设、企业发展和群众创业成本。坚持严管厚爱，奖优治庸罚懒，坚决解决行政不作为、乱作为和推诿扯皮问题，坚决查处吃拿卡要、中梗阻、破坏经济发展环境的案件。讲诚信，凡政府承诺的事情，坚决兑现，绝不失信于民。

　　坚持依法行政。严格按照法定权限和程序行使权力、履行职责。自觉接受人大法律监督、工作监督和政协民主监督，接受社会和新闻舆论监督。进一步完善社会公示、新闻发布、重大决策听证和专家咨询制度，认真听取和吸纳社会各界的意见和建议，改进政府工作，坚持科学决策、民主决策。

　　加强廉政建设。坚持教育、制度、监督、改革、纠风、惩治相结合，推进惩治和预防腐败体系建设，从严治政。认真落实党风廉政建设责任制，采取有力措施，切实纠正损害群众利益的突出问题。坚持权为民所用、情为民所系、利为民所谋，模范遵守廉洁从政各项规定，建设廉洁政府。

　　各位代表！今天的四平正处在一个夯实基础、积蓄能量、快速崛起的关键时期，“四平振兴、人民富裕”的目标等待我们去完成。困难和挑战考验着我们，责任和使命激励着我们。让我们在市委的领导下，开拓进取，扎实工作，为开创四平经济社会发展的新局面而努力奋斗！
